# A Mendelian randomization study to examine the causal associations of circulating micronutrient levels with frailty risk

**DOI:** 10.3389/fnut.2024.1386646

**Published:** 2024-04-30

**Authors:** Kaidiriyan Kuribanjiang, Yiping Min, Shikang Yan, Simin Chen, Nuerbiyamu Aiheti, Abudunaibi Wupuer, Jie Wang, Xing Peng, Yihan Li, Huilong Li, Yiran Dong, Yuanlin Fan, Lei Yang, Jianping Zhao

**Affiliations:** ^1^Department of Epidemiology and Health Statistics, School of Public Health, Xinjiang Medical University, Ürümqi, Xinjiang, China; ^2^College of Mathematics and System Science, Xinjiang University, Ürümqi, Xinjiang, China; ^3^State Key Laboratory of Causes and Prevention of High Morbidity in Central Asia Jointly Established by the Ministry and the Province Urumqi, Ürümqi, Xinjiang, China

**Keywords:** circulating micronutrients, frailty, genome-wide association studies, Mendelian randomization, meta-analyses

## Abstract

**Background:**

Observational studies have shown that micronutrients can affect the occurrence of frailty. However, it is not clear whether there is a causal relationship between the two. This study aimed to explore the causal relationship between circulating micronutrient levels and frailty risk using a two-sample Mendelian randomization (TSMR) approach.

**Methods:**

We gathered and screened instrumental variables (IVs) for six circulating micronutrients, including vitamin B_12_, vitamin B_6_, folate, vitamin C, vitamin D, and vitamin E, from published genome-wide association studies (GWAS) and the IEU OpenGWAS open database. Summary statistics for frailty were obtained from a GWAS meta-analysis, including the UK Biobank and TwinGene (*N* = 175,226). We performed two independent TSMR analyses and a meta-analysis based on the two independent MR estimates to assess the causal relationship between circulating micronutrientn and frailty.

**Results:**

Our study found, no causal relationship between genetically predicted vitamin D (*β* = −0.059, *p =* 0.35), vitamin B_6_ (*β* = 0.006, *p =* 0.80), vitamin E (*β* = −0.011, *p =* 0.79), vitamin C (*β* = −0.044, *p =* 0.06), vitamin B_12_ (*β* = −0.027, *p =* 0.37), and folate (*β* = 0.029, *p =* 0.17), with frailty.

**Conclusion:**

This study showed that these six micronutrients did not reduce the risk of developing frailty. However, we think it is necessary further to investigate the relationship and mechanisms between micronutrients and frailty using methods such as randomized controlled trials.

## 1 Introduction

Frailty has become a serious health concern as the world’s population ages (1, 2). Frailty is essentially described as an age-related reduction in physiologic reserve and function across multiple organ systems that diminishes the body’s capacity to deal with daily or acute stresses (3–5). Frailty has been connected to poor health outcomes that involve an increased chance of falling, disability, hospitalization, and death, and it puts a more significant strain on society’s health care resources (6–8). Based on how it is measured, frailty prevalence varies. Recent studies indicated that using the phenotypic frailty evaluation, the prevalence of frailty in the population was 12%, but the predicted prevalence of frailty based on the Frailty Index (FI) was roughly 24% (9–12). Frailty is reversible and may be prevented by altering its risk factors.

With age, gastric motility, and gastrointestinal hormones change (13), which can alter dietary preferences and intake as well as affect macro- and micronutrient absorption. As a result, older persons are more susceptible to protein-energy and micronutrient malnutrition. Of these, the consequences of micronutrient deficiencies are sometimes permanent (14). Epidemiological studies have shown a correlation between micronutrients and frailty. Xiong et al. found, a negative association between vitamin D and risk of frailty (15). In addition, a study by Das et al. showed that low vitamin E intake was significantly associated with a high prevalence of frailty (16). A cross-sectional survey from Japan identified that folate and vitamin C were significantly associated with frailty status in men (17). Cheng et al. conducted a study on vitamin B intake and risk of frailty in chronic obstructive pulmonary disease (COPD) patients and found (18) that COPD patients with lower vitamin B6 intake have a higher risk of frailty. The a cross-sectional study of Soh et al. have shown, low vitamin B_12_ in the blood increased the incidence of frailty (19). However, observational studies are susceptible to confounding factors, which may lead to misestimation of the actual association between exposure and outcome. Thus, the existence of a causal relationship between circulating micronutrients and frailty is unclear, and a methodology that can account for causality is needed to explore the relationship between them.

Mendelian randomization (MR) is an approach for determining the causal relationship between risk variables and disease (20). MR effectively mitigates the influence of confounding factors via the use of genetic differences as IVs (20). The random assignment of alleles influencing genetic variants during conception, unaffected by environmental factors or unknown confounding variables, allows for MR analysis that resembles naturally occurring randomized controlled trials. Thus, this approach enables the assessment of connections between exposure and outcome at the genetic level while eliminating the potential for reverse causality (20, 21). Nevertheless, there has been a shortage of research that uses MR analysis to investigate the connection between micronutrients and frailty.

This study used the two-sample Mendelian randomization (TSMR) method to investigate, the causal relationship between micronutrients of vitamin B_12_, vitamin B_6_, folate, vitamin C, vitamin D, and vitamin E with frailty, designed to provide new insights into the prevention of frailty.

## 2 Methods

### 2.1 Study design

**Figure 1:**
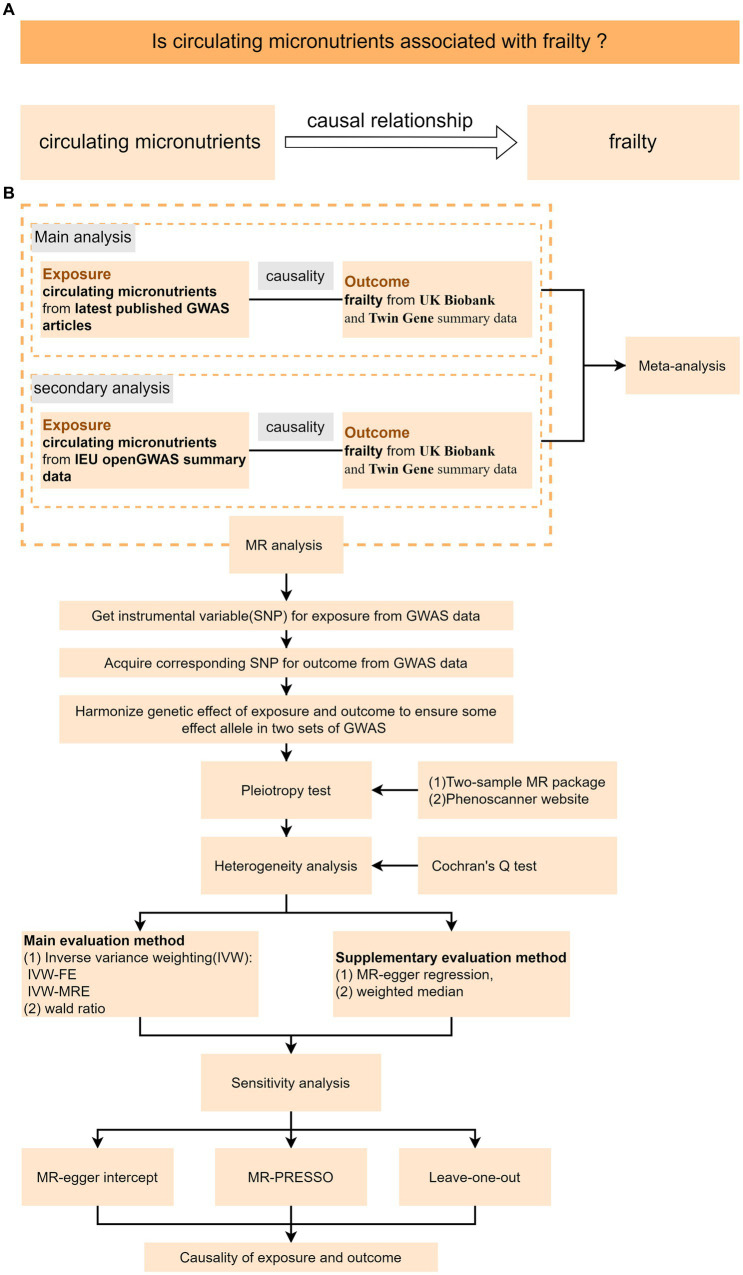
Schematic diagram of the TSMR study. **(A)** MR analysis’s research design. **(B)** MR analysis’s flow chart; IVW-FW stands for fixed for fixed effect inverse variance weighted model, while IVW-MRE stands for multiple inactive random-effect inverse variance weighted model.

In this study, we used circulating micronutrients as an exposure variable and frailty as an outcome variable, conducted a TSMR Investigating the probable causative association between the six micronutrients (vitamins B_12_, B_6_, C, D, E, and folate) with frailty (Figure 1A). We performed MR analysis using published genome-wide association studies (GWAS). After that, we performed a secondary analysis using the OpenGWAS database. Finally, a meta-analysis was performed on the two MR results. The flowchart of the design of this study is shown in Figure 1B. This MR investigation met the three basic assumptions described in Supplementary Figure S1. Based on the first assumption (Relevance), IVs were strongly linked with exposure. According to assumption 2 (Independence), IVs must be free of any confounders related to exposure and outcome. Based on Assumption 3 (Exclusivity), IVs can only affect outcomes via exposure. This research used publicly accessible data, thereby obviating the need for ethical clearance and informed consent.

### 2.2 Exposure data sources

The IEU OpenGWAS database^1^ and PubMed^2^ were searched for published GWAS examining people with European ancestry (the most recent search was done in May 2023). Vitamins B_12_, B_6_, C, and folate were obtained from published GWASs, while vitamin D data were obtained from the SUNLIGHT Consortium and vitamin E data from IEU OpenGWAS. Detailed information on exposure data is provided in Table 1.

**Table 1:** Main analysis exposure source – summary data from GWAS study.

Exposures	Cohorts or datasets	Participants	Publicly available websites	PMID
Vitamin C (22)	Fenland study, EPIC InterAct study, EPIC Norfolk study, EPIC-CVD study	52,018 individuals of European ancestry	https://doi.org/10.6084/m9.fifigshare.13227443.v1	33,203,707
Vitamin B_12_ (23)	Icelanders, Danish-Inter99, Danish-Health2006	45,576 individuals of European ancestry	NA	23,754,956
Folic acid (21)	Icelanders,Danish-Inter99, Danish-Health2006	37,341 individuals of European ancestry	NA	23,754,956
Vitamin B_6_ (24)	CGEMS study, SHARe study	4,763 individuals of European ancestry	NA	19,744,961
Vitamin D	Summary statistics of the meta-analysis between the UKB 25OHD GWAS and the SUNLIGHT consortium GWAS.	417,580 individuals of European ancestry	https://jrevez.github.io/vitaminD/index.html	32,242,144
Vitamin E (25)	The Twins UK cohort and the KORA study	7,725 individuals of European	https://gwas.mrcieu.ac.uk//datasets/met-a-340/	24,816,252

### 2.3 Outcome data sources

For this study, frailty data were obtained from the IEU openGWAS database. This study used data from two different cohorts: European-descent UK Biobank participants (*n* = 164,610, ages 60 to 70 years) and Swedish Twin Gene participants (*n* = 10,616, ages 41 to 87 years), and used the frailty index (FI) to measure frailty (26). Of these, the Frailty Index combines dozens of parameters, including symptoms, signs, disabilities, and diagnosed disease items, and reflects the accumulation of potential health deficits throughout, for life.

### 2.4 Instruments selection

A meta-analysis using four independent investigations with more than 50,000 individuals discovered 11 genetic variants related to circulating vitamin C (*p* < 5.0 × 10^−8^). A GWAS encompassing 82,917 participants discovered five genetic variants for circulating vitamin B_12_ levels and two genetic variants for circulating folate levels (linkage disequilibrium parameter (r^2^) < 0.001; *p* < 5 × 10^−8^). Two genetic variables related to vitamin B_6_ were found in a meta-analysis of two separate investigations with 4,763 individuals (*p* < 5 × 10^−8^; r^2^ < 0.001). For 25-hydroxyvitamin D and vitamin E, we set the *p*-value, r^2^, and genetic distance. The *p*-value for 25-hydroxyvitamin D was less than or equal to 5 × 10^−8^, and the *p*-value for vitamin E was less than or equal to 5 × 10^−6^, r^2^ = 0. 001, and the genetic distance was 10,000 kb. To exclude weak instrumental variables, we calculated the F-statistics of the IVs, which are generally required to be *F* > 10 (27). Finally, 120 SNPs related to six micronutrients were selected. Detailed information on these SNPs can be found in Supplementary Table S1.

### 2.5 Analysis of horizontal pleiotropy and heterogeneity

Initially, we achieved harmonization of the exposure data and outcome data through the process of matching the SNPs. Furthermore, it is essential to consider that the pleiotropy of SNPs in the IVW research, which may introduce bias and distort causal estimates and conclusions (28). Therefore, to check if the SNPs were acceptable IVs, we utilized the pleiotropy test function with the two-sample MR package (29). The IVs could be used if the pleiotropy was insignificant (*p* > 0.05). If the pleiotropy test’s *p*-value was less than 0.05, we manually chose each SNP on the Phenoscanner website (30). After that, we used Cochran’s Q to assess the heterogeneity. In situations where the degree of heterogeneity was assessed to be negligible, the fixed effects model was used. In contrast, a random effects model was considered required if heterogeneity was present.

### 2.6 Analysis of Mendelian randomization

For each SNP, we determined the Wald ratio, the ratio of the connection between the SNP’s exposure and outcome. The Wald ratio estimate approach was used for MR analysis when a micronutrient had only one SNP. We explored the causal relationship between micronutrients and frailty for micronutrients containing multiple SNPs using the inverse-variance weighted (IVW) method, which was also the primary method used in this study, by combining the Wald ratios of the causal effects of each SNP to estimate the causal effect. We also conducted additional analyses using the weighted median and MR-Egger methods to help further confirm the findings.

### 2.7 Sensitivity analysis

We used the MR-Egger intercept, the MR-Pleiotropy RESidual Sum and Outlier (MR-PRESSO), and the leave-one-out approach as sensitivity tests to evaluate the findings, further to ensure the accuracy of the MR causal effect estimation. To assess if SNPs showed horizontal pleiotropy, the MR-Egger method was used (31). The IVs could possess pleiotropic effects if the *p*-value for the intercept term were less than 0.05. However, if the *p*-value of the intercept term was more extensive than 0.05, there was no proof of horizontal pleiotropy across the selected IVs. The MR-PRESSO test, which provided a reliable estimate after outlier correction, was used to identify the typical MR testing outliers (32). Finally, we using the leave-one-out method to determined whether outliers could be skewing the overall MR estimate.

### 2.8 Secondary analyses

Exposure data for secondary analyses were derived from the IEU OpenGWAS database (see Table 2 for details). In the secondary analysis, the criterion for selecting IV was relaxed, i.e., the *p*-values of all six micronutrients were less than 5 × 10^−6^, and the linkage disequilibrium (LD) was consistent with the primary analysis criterion. While doing so could boost statistical power, there is also a chance that the MR assumptions were broken and weak instrument bias introduced. Therefore, the F statistic was calculated, and SNPs with *F* < 10 were excluded. Finally, 257 SNPs related to six micronutrients were selected, and the specific information on SNPs is shown in Supplementary Table S2.

**Table 2:** Source of secondary analysis exposure – genome-wide association study summary data.

Phenotype	Consortium	Participants	Datatype	GWAS ID	Year of publication
Vitamin C	MRC-IEU	64,979individuals of European ancestry	Continuous	ukb-b-19390	2018
Vitamin B_12_	MRC-IEU	64,979individuals of European ancestry	Continuous	ukb-b-19524	2018
Folate acid	MRC-IEU	64,979individuals of European ancestry	Continuous	ukb-b-11349	2018
Vitamin B_6_	UKB	64,979individuals of European ancestry	Continuous	ukb-b-7864	2018
Vitamin D	UKB	443,734individuals of European ancestry	Continuous	ebi-a-GCST010144	2020
Vitamin E	UKB	64,979individuals of European ancestry	Continuous	ukb-b-6888	2018

### 2.9 Meta-analysis

We conducted a meta-analysis of the findings of the main and secondary investigations to investigate further the causative influence of genetically circulating micronutrients on the risk of frailty. To assess heterogeneity, we employed the Q test and the inconsistency (I^2^) test. If I^2^ was more than 50% and *p* < 0.05, heterogeneity was considered (33). We utilized a fixed-effects model for the meta-analysis if there was no heterogeneity; otherwise, we used a random-effects model.

### 2.10 Reported results and software

For dichotomous variables, the MR findings were shown as odds ratios (ORs) and 95% confidence intervals (CIs) per standard deviation. The results were reported for continuous variables as beta values (*β*) and 95% CIs per standard deviation. Considering that the MR analysis in this study involved sex exposures, the *p*-value was corrected for Bonferroni, and the corrected *p*-value was calculated to be 0.00833 (0.05/6, 6 being the number of tests). That is, *p* < 0.00833 is evidence of causation, while 0.00833 < *p* < 0.05 is suggestive evidence of causation. The meta-analyses and MR analyses were conducted using the “meta” package, the “TwoSampleMR” package, and the “MRPRESSO” package, all of which are based on the R programming language.

## 3 Results

### 3.1 Main analysis

Pleiotropy and heterogeneity tests on the selected IVs revealed no pleiotropic SNPs. In addition, we observed significant heterogeneity for vitamin D and C (Q = 214.306, *p*-value = 1.726 × 10^−10^; Q = 28.792, *p*-value =0.001) but no heterogeneity for the remaining four micronutrients. Therefore, we used IVW in randomized mode to obtain the effect value of vitamin D and C. Supplementary Table S3 provides further details regarding this finding.

**Figure 2:**
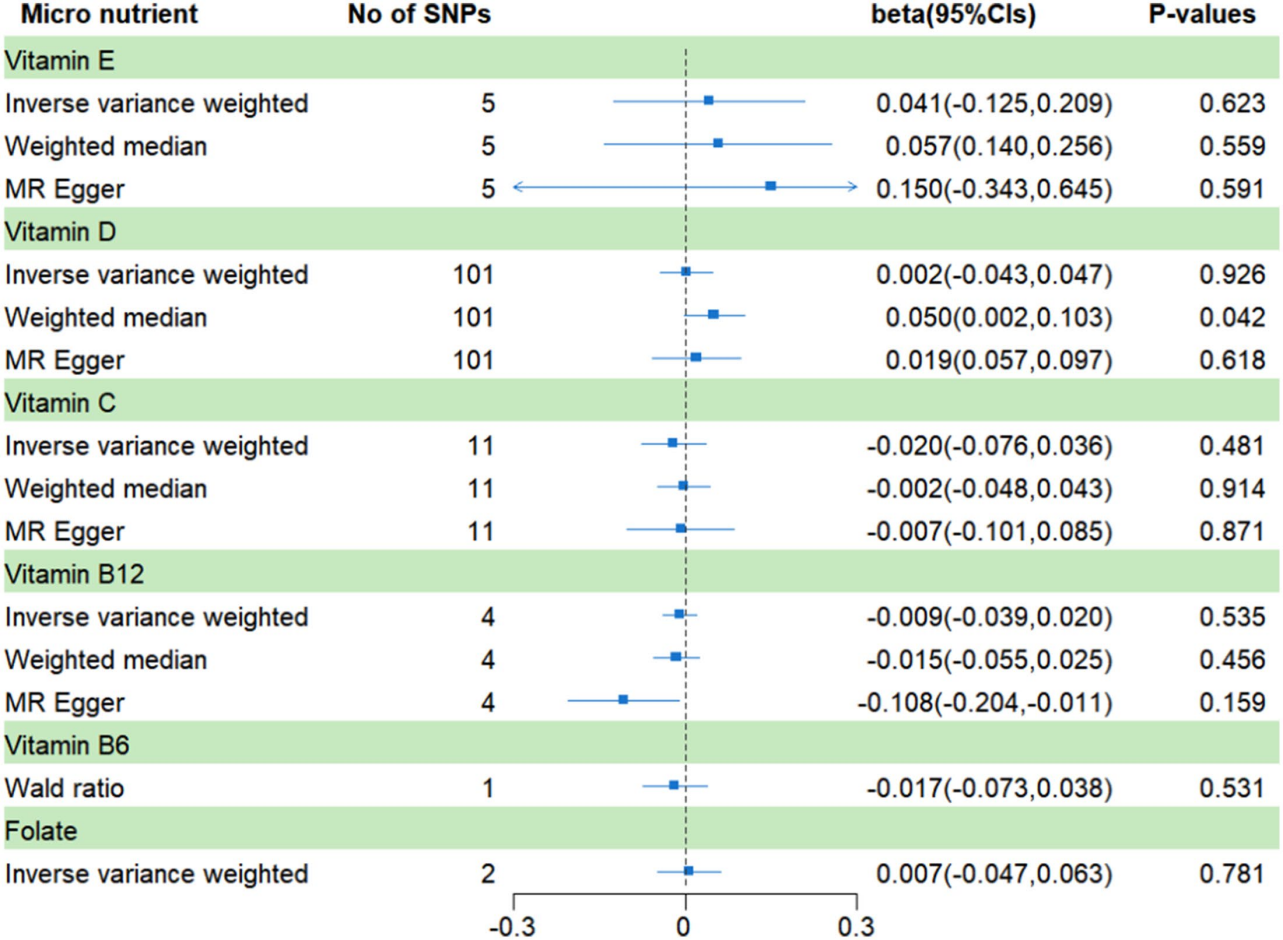
MR analysis of genetically predicted levels of circulating micronutrients and frailty risk in main analysis.

As shown in Figure 2, the MR estimates obtained by the IVW method suggested that the predicted circulating levels of six micronutrients, including vitamin C (*β* = −0.02; 95% CI: −0.076, 0.036; *p* = 0.481), vitamin B_12_ (*β* = −0.009; 95% CI: −0.039, 0.020; *p* = 0.535), folate (*β* = 0.007; 95% CI: −0.047, 0.063; *p* = 0.781), vitamin D (*β* = 0.002; 95% CI: −0.043, 0.047; *p* = 0.926), and vitamin E (*β* = 0.041; 95% CI: −0.125, 0.209; *p* = 0.623), were causally unrelated to frailty. The weighted median method showed a suggestive causal association between vitamin D and frailty (*β* = 0.050; 95% CI: 0.002, 0.103; *p* = 0.042), while the remaining four vitamins yielded similar results to IVW. Additionally, neither the MR-Egger intercept nor the MR-PRESSO method revealed any apparent horizontal pleiotropic effects associated with these SNPs (see Supplementary Table S4). The leave-one-out analysis conducted in this study did not identify any SNP that had a significant influence on the results of any MR studies. Supplementary Figures S2–S5 provide further details. For vitamin B_6_, which had only one SNP as an instrumental variable, we analyzed its association with frailty using the Wald ratio method, and the results did not provide any evidence of a causal relationship between vitamin B_6_ and frailty (*β* = −0.017; 95% CI: −0.073, 0.038; *p* = 0.531).

### 3.2 Secondary analysis

Pleiotropy and heterogeneity tests on the selected IVs revealed no pleiotropic SNPs. Further, we observed significant heterogeneity for vitamin E and D (Q = 23.015, *p*-value =0.017; Q = 363.67, *p*-value = 4.990 × 10^−12^) but no heterogeneity for the remaining four micronutrients. Therefore, we used IVW in randomized mode to obtain the effect value of vitamin E and D. Supplementary Table S5 provides further details regarding this finding.

**Figure 3:**
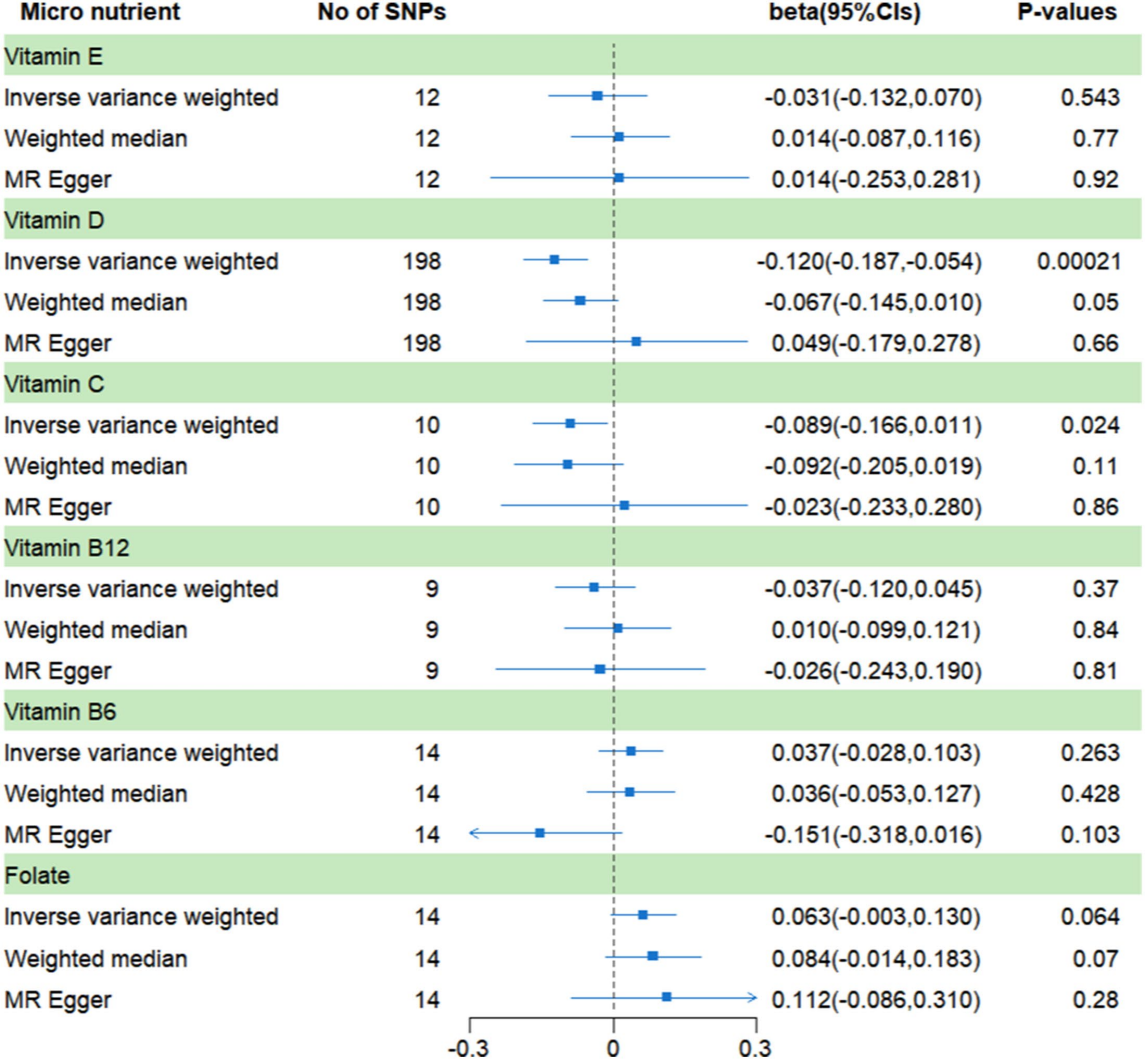
MR analysis of genetically predicted levels of circulating micronutrients and frailty risk in secondary analysis.

In the secondary analysis (Figure 3), a significant causal relationship was found between vitamin D and frailty, vitamin C has a suggestive causal association with frailty. The *β* values for vitamin D and vitamin C were − 0.12 (95% CI: −0.187, −0.054; *p* = 0.00021) and − 0.089 (95% CI, −0.166, 0.011; *p* = 0.024), respectively. The results regarding the remaining four micronutrients were found to be consistent with the results of the main analysis, showing no significant causal relationships with frailty. Furthermore, there were no noticeable horizontal pleiotropic effects associated with these SNPs according to the MR-Egger intercept and MR-PRESSO approaches. Supplementary Table S6 provides further details. The leave-one-out analysis conducted for all MR studies did not identify any SNP that had a significant influence on the results (see Supplementary Figures S6–S11).

### 3.3 Meta-analysis

**Figure 4:**
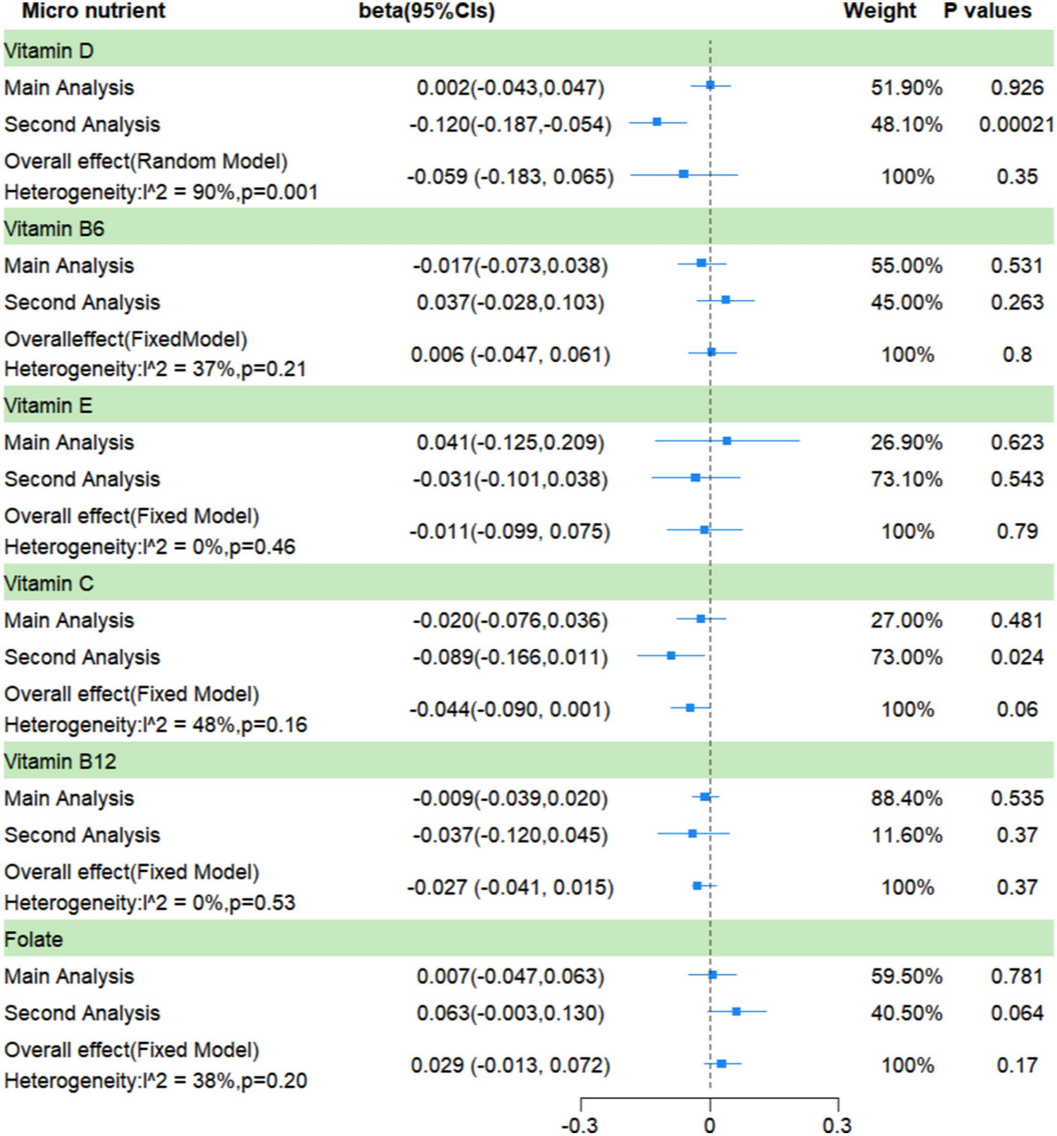
A meta-analysis based on the results of two Mendelian randomization analyses.

In this part, vitamin D had significant heterogeneity so it was analyzed with a random-effects model; the rest used a fixed-effects model. And, the results show, that there was no conclusive evidence of a causal connection between the circulating levels of the six micronutrients and frailty. For the circulating levels of the six micronutrients, the pooled *β*s are −0.044 (vitamin C, 95% CI: −0.090, 0.001; I^2^ = 48%, *p* = 0.06), −0.027 (vitamin B_12_, 95% CI: −0.041, 0.015; I^2^ = 0%, *p* = 0.37), 0.029 (folate, 95% CI: −0.013, 0.072; I^2^ = 38%, *p* = 0.17), −0.059 (vitamin D, 95% CI: −0.183, −0.065; I^2^ = 90%, *p* = 0.35), −0.011 (vitamin E, 95% CI: −0.099, 0.075; I^2^ = 0%, *p* = 0.79), and 0.006 (vitamin B_6_, 95% CI: −0.047, 0.061; I^2^ = 37%, *p* = 0.80) (Figure 4).

## 4 Discussion

The current research demonstrated no evidence of a significant causal connection between the genetically predicted circulating levels of vitamins B_6_, B_12_, E, D, and C, and folate with the risk of frailty.

Recent research has revealed a strong correlation between elevated homocysteine levels with inflammation, mitochondrial dysfunction, and oxidative stress, which can result in muscle loss, diminished physical performance, and, ultimately, frailty (34). And studies have found, that vitamins B_6_, B_12_ and folate may alter homocysteine status through regulation of single-carbon metabolism (35). Thus, several studies have analyzed the possibility that these three B vitamins contribute to the frailty. For instance, a Spanish study found an association between a greater risk of frailty and a lower consumption of vitamin B_6_ and folate (13). According to Matteini et al. vitamin B_12_ deficiency in older women who live in the community may exacerbate the frailty syndrome (36). The prospective findings of Semba et al. have shown, however, that folate and vitamin B_6_ were not substantially related to the incidence of frailty (37). Furthermore, a cross-sectional study conducted in Korea showed that the risk of frailty and circulating vitamin B_12_ levels were not correlated (19). All in all, discrepancies in the outcomes of the three micronutrient studies discussed above might be attributed to variances in research sample sizes, vulnerability assessment approaches, and laboratory testing operations. Thus, we used the more trustworthy MR analysis and ultimately concluded that there was no causal connection between the risk of frailty and the genetically predicted circulating levels of vitamins B_12_, B_6_, and folate.

Vitamins E and C, the most prevalent antioxidants (16), may have the ability to prevent the onset and development of frailty by lowering oxidative stress. The observational studies, suggesting that vitamins C and E may be related to a lower incidence of frailty (16, 38). Besides that, a part of the study has investigated their relationship with frailty phenotypes such as skeletal muscle mass and grip strength. Lewis et al. examined the relationship between dietary and circulating vitamin C with skeletal muscle mass, and found a positive correlation between vitamin C with skeletal muscle mass (39). In addition, from a study analyzing the association between the NHANES cohort’s dietary antioxidant and grip strength, vitamin E intake was found to be positively associated with grip strength in men (40). In contrast, a study conducted in Switzerland demonstrated that the supplementation of vitamins C and E neither improved nor prevented low grip strength over a five years (41). Our MR analyses showed no causal association between circulating vitamin E and C levels and risk of frailty.

As the study of vitamin D has intensified, researchers have found a correlation between it and autism (42), depression (43), and cognitive impairment (44). Numerous observational studies have also analyzed the association between vitamin D and the risk of frailty. However, to date, the results of these studies have been inconsistent. Some of the researchers think that vitamin D deficiency may contribute to frailty by decreasing muscle function (45). In addition, since both immune activation and chronic inflammation have been implicated in the etiology of frailty (46), it is hypothesized that the anti-inflammatory effects of vitamin D may be another mechanism. An observational study from South Korea showed that in older persons who live in the community, low levels of vitamin D have been associated with an increased risk of frailty (47). However, the prospective findings of Buta et al. showed that, after adjusting for the cardiometabolic disease, low circulating vitamin D levels were not considerably correlated with the risk of frailty (48). There was also no statistically significant relationship between circulating 25-hydroxyvitamin D levels and frailty, according to Krams T. et al.’s investigation of 321 individuals (49). Therefore, we introduced MR analysis in the hope of obtaining more reliable results. Our findings do not support a causal connection between genetically predicted circulating levels of vitamin D and the occurrence of frailty.

This study has the following advantages. First, our research is the first known MR study to evaluate different micronutrients and the risk of becoming frail. Second, the instrumental variables employed in our study were sourced from recently published articles. Additionally, we received summary information on frailty from the IEU OpenGWAS database, which has a large sample size of over 270,000 people and is, therefore, better able to establish a causal connection between the exposure and the outcome. Finally, utilizing information from separate GWAS studies and public databases, respectively, we examined the causal connection between micronutrients and frailty. We then meta-analyzed the findings of the two MRs, and the concordance of these results supported our findings.

We must admit that our research had significant limitations. First, because the GWAS pooled data on frailty originated from a European population, it is necessary to conduct further research to determine whether the findings of this study can be generalized. Second, our capacity to conduct subgroup analyses was constrained since the frailty data we utilized could not be classified according to variables (age, sex, smoking, alcohol use, underlying disorders, or deficits of certain micronutrients in the population). Third, it should be noted that residual bias remains an inherent limitation of the MR approach, even when using pleiotropy tests and MR-PRESSO methods to mitigate the potential confounding effects caused by pleiotropy.

## 5 Conclusion

In conclusion, there was no indication of a causal connection between genetically predicted circulation levels of six micronutrients and frailty risk in this MR research. The TSMR study could not to detect nonlinear causation; nonetheless, nonlinear causality between circulating micronutrient levels and frailty risk might exist. As a result, more sophisticated methodologies, such as nonlinear Mendelian randomization research, are required to identify possible causal correlations between micronutrients and frailty risk. In addition, we hope that future researchers will be able to use methods, such as randomized controlled trials and animal experiments to analyze the relationship and possible mechanisms between each micronutrient and frailty, thus providing a more reliable basis for future research.

## Data availability statement

The original contributions presented in the study are included in the article/Supplementary material, further inquiries can be directed to the corresponding authors.

## Author contributions

KK: Writing – original draft, Writing – review & editing. YM: Data curation, Methodology, Writing – original draft. SY: Methodology, Writing – review & editing. SC: Data curation, Writing – review & editing. NA: Conceptualization, Writing – review & editing. AW: Data curation, Writing – review & editing. JW: Software, Writing – review & editing. XP: Conceptualization, Writing – review & editing. YL: Investigation, Writing – review & editing. HL: Visualization, Writing – review & editing. YD: Software, Writing – review & editing. YF: Validation, Writing – review & editing. LY: Supervision, Writing – review & editing, Funding acquisition. JZ: Supervision, Writing – review & editing.

## Footnotes

^1^ https://gwas.mrcieu.ac.uk/

^2^ https://www.ncbi.nlm.nih.gov/pubmed
