# Supplementary material for: A Mendelian randomization study to examine the causal associations of circulating micronutrient levels with frailty risk
Source: Front Nutr. 2024 Apr 30;11:1386646. doi: 10.3389/fnut.2024.1386646 (PMC11091248; doi:10.3389/fnut.2024.1386646)
Supplement: Supplementary file 1 [file Data_Sheet_1.PDF]

Supplementary Material

1 Supplementary Figures and Tables

1.1 Supplementary Figures

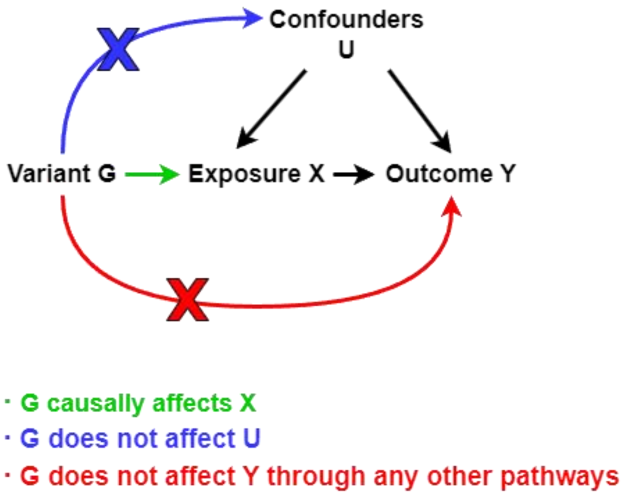

Supplementary Figure 1. Our MR analysis's fundamental schema

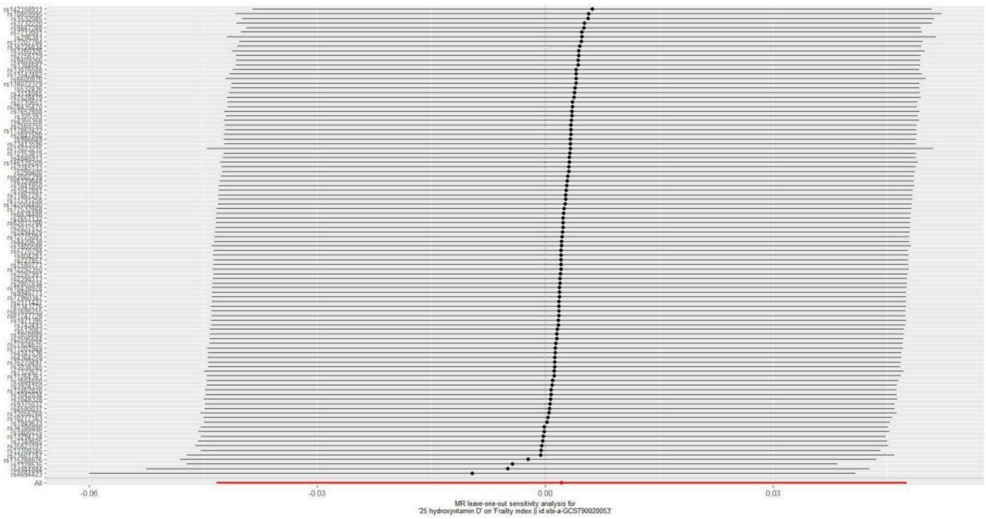

Supplementary Figure 2. The leave-one-out method of analysis of frailty and vitamin D in the main analysis.

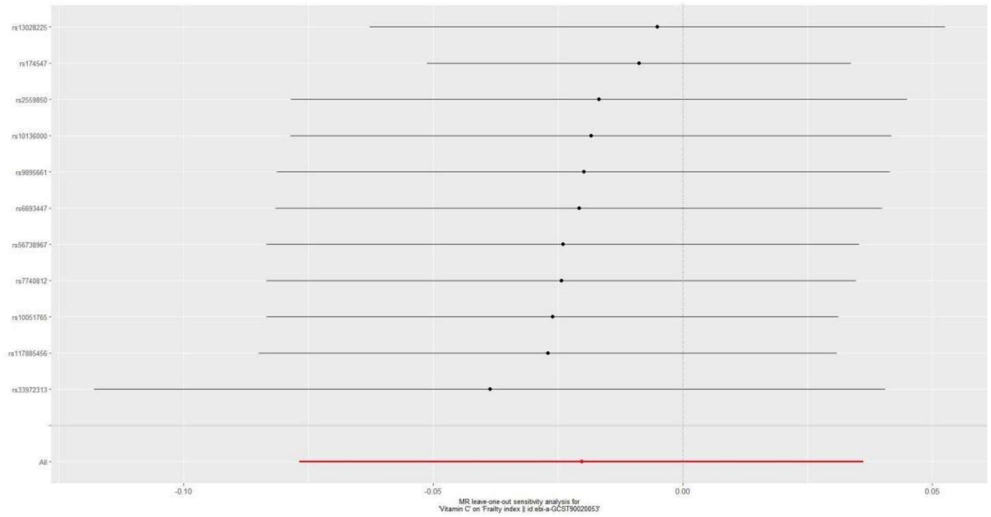

Supplementary Figure 3. The leave-one-out method of analysis of frailty and vitamin C in the main analysis

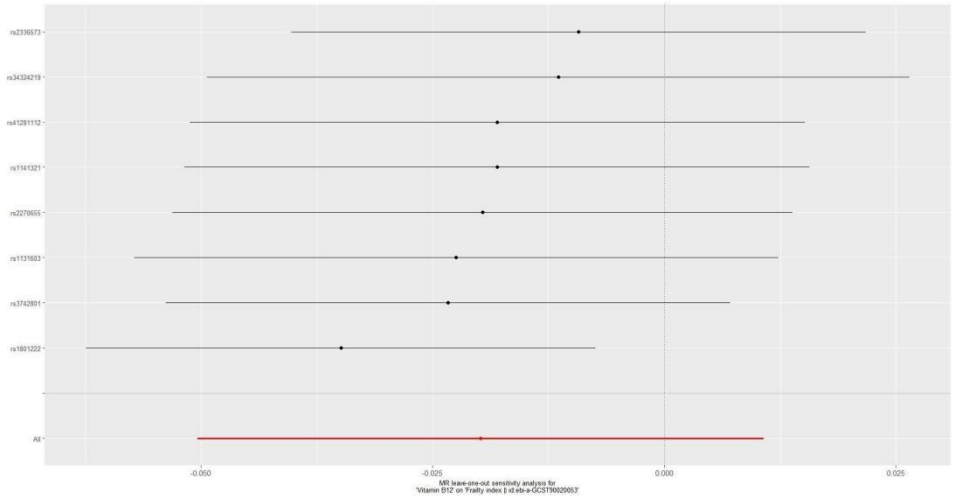

Supplementary Figure 4. The leave-one-out method of analysis of frailty and vitamin B_{12} in the main analysis

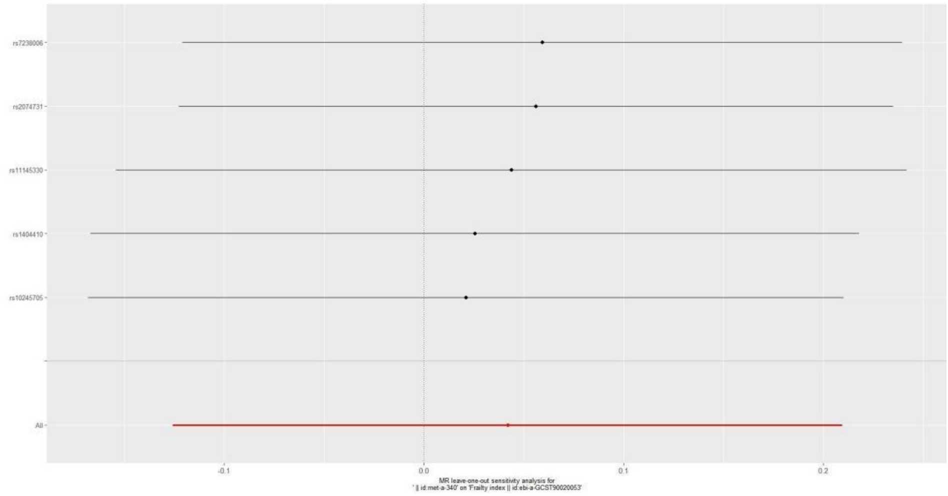

Supplementary Figure 5. The leave-one-out method of analysis of frailty and vitamin E in the main analysis

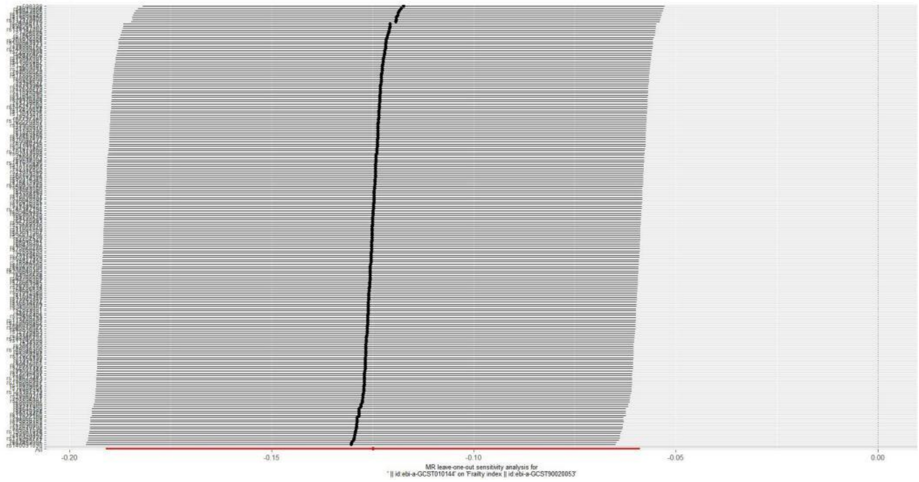

Supplementary Figure 6. The leave-one-out method for the analysis of frailty and vitamin D in secondary analysis

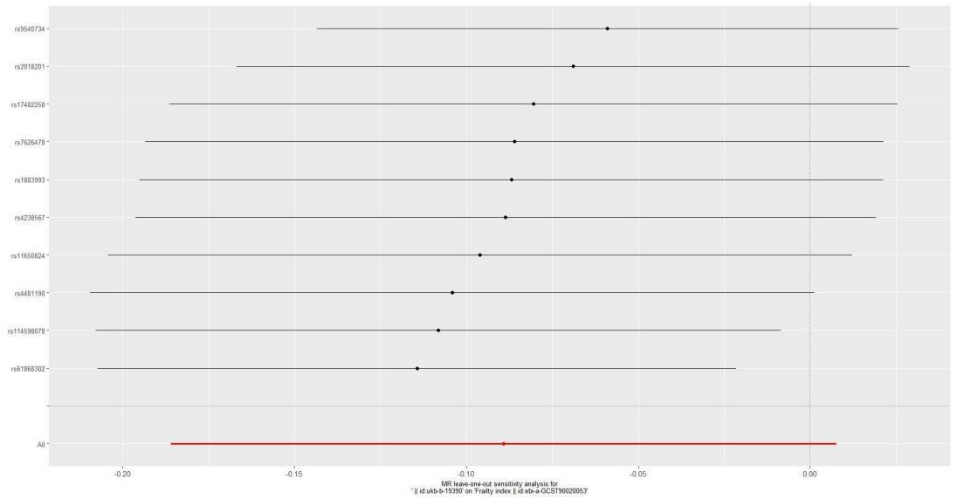

Supplementary Figure 7. The leave-one-out method for the analysis of frailty and vitamin C in secondary analysis

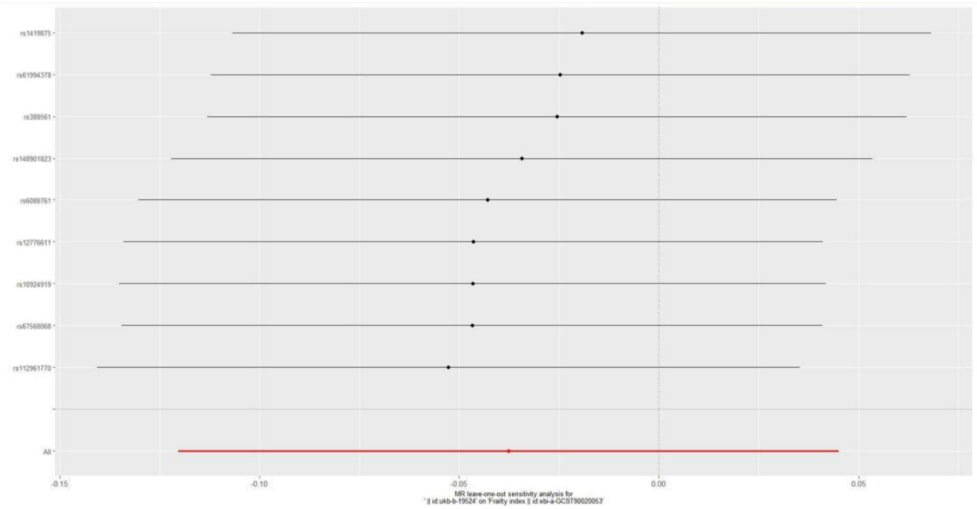

Supplementary Figure 8. The leave-one-out method for the analysis of frailty and vitamin B_{12} in secondary analysis

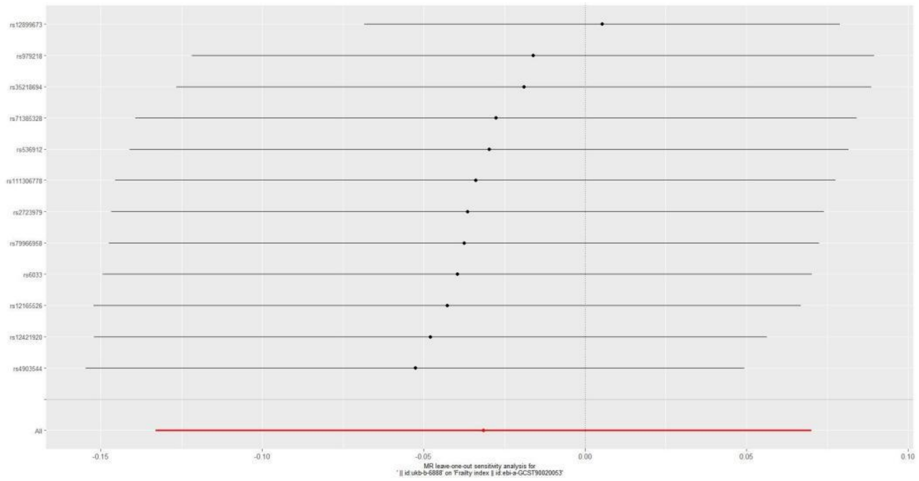

Supplementary Figure 9. The leave-one-out method of analysis of frailty and vitamin E in the secondary analysis

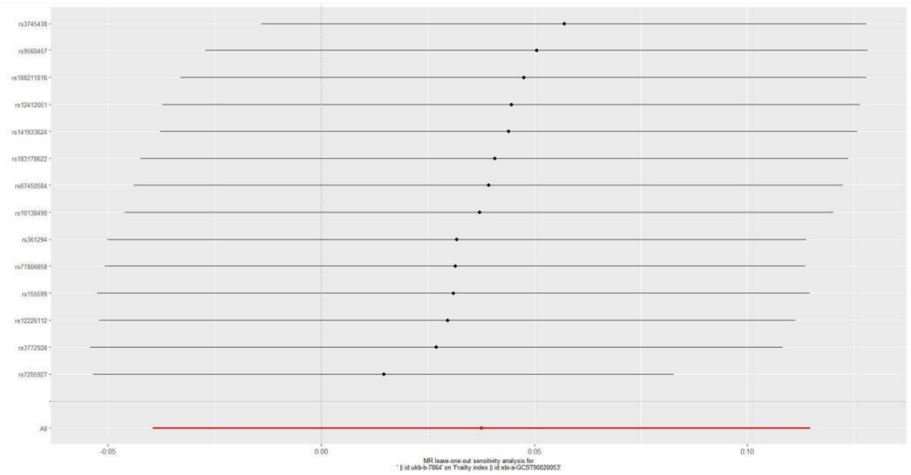

Supplementary Figure 10. The leave-one-out method for the analysis of frailty and vitamin B_6 in secondary analysis

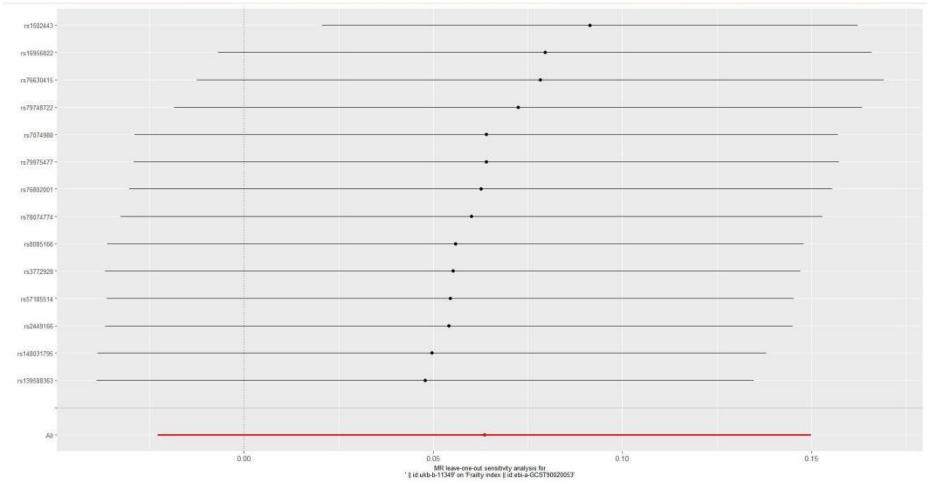

Supplementary Figure 11. The leave-one-out method for the analysis of frailty and folate in secondary analysis

1.2 Supplementary Tables

Supplementary Table 1. Instrumental variables for main analysis screening

Micro nutrient	SNPs	EA	OA	BETA	SE	P-vale	F
Vitamin D	rs10277163	G	A	-0.0143	0.0024	1.08E-09	37.181
	rs1038165	C	T	-0.0115	0.0021	2.15E-08	31.358
	rs1042034	C	T	0.0151	0.0025	1.45E-09	36.599
	rs10438978	T	C	0.0172	0.0026	7.34E-11	42.427
	rs1047891	A	C	-0.0134	0.0022	7.96E-10	37.769
	rs1048328	A	G	0.0313	0.0037	5.58E-17	70.121
	rs10859995	T	C	0.0436	0.0021	4.60E-100	450.898
	rs11023215	A	G	-0.0643	0.0021	1.00E-200	924.263
	rs11207969	G	A	0.0209	0.0021	7.14E-23	96.943
	rs11264361	G	T	0.0175	0.0023	7.97E-14	55.812
	rs1128535	C	T	-0.0164	0.0020	6.06E-16	65.416
	rs1149605	C	T	0.0225	0.0027	1.30E-16	68.445
	rs115288876	A	G	0.0788	0.0050	2.36E-56	250.188
	rs11542462	A	G	-0.0248	0.0030	9.72E-17	69.025
	rs117862422	C	T	-0.0505	0.0087	5.52E-09	33.997
	rs11791258	A	G	0.0141	0.0026	4.85E-08	29.774
	rs11867297	T	C	0.0135	0.0021	1.01E-10	41.809
	rs12056768	T	G	0.0232	0.0021	2.65E-29	126.294
	rs12153819	T	C	-0.0178	0.0031	8.16E-09	33.237

	rs12292350	T	C	-0.0348	0.0038	6.01E-20	83.617
	rs12462826	A	G	-0.0132	0.0021	4.18E-10	39.025
	rs1260326	T	C	-0.0197	0.0021	1.96E-21	90.382
	rs12775091	T	C	0.0156	0.0025	3.33E-10	39.472
	rs13076508	C	T	0.0251	0.0045	2.78E-08	30.856
	rs13108245	G	A	-0.0122	0.0021	4.63E-09	34.338
	rs13294734	T	C	0.0126	0.0021	1.02E-09	37.281
	rs1343776	A	G	0.0181	0.0025	1.62E-13	54.423
	rs138072379	T	C	0.0443	0.0073	1.29E-09	36.835
	rs1384687	A	G	-0.0169	0.0030	1.82E-08	31.674
	rs142004400	C	A	-0.0310	0.0056	3.01E-08	30.699
	rs142158911	A	G	0.0263	0.0032	4.43E-16	66.036
	rs146128209	G	A	-0.0484	0.0040	2.61E-34	149.189
	rs1532085	A	G	-0.0253	0.0021	8.60E-34	146.818
	rs1684600	T	C	-0.0125	0.0022	1.59E-08	31.938
	rs17207784	C	T	-0.0135	0.0022	5.14E-10	38.622
	rs1800588	T	C	-0.0305	0.0025	4.73E-35	152.583
	rs1800775	A	C	-0.0174	0.0020	9.23E-18	73.671
	rs1841850	C	A	0.0304	0.0032	6.73E-22	92.502
	rs1858889	A	C	-0.0135	0.0020	3.49E-11	43.880
	rs1871395	G	A	-0.0204	0.0028	5.72E-13	51.940
	rs1949633	T	C	-0.0114	0.0021	4.45E-08	29.941
	rs2037511	A	G	0.0177	0.0027	9.41E-11	41.940
	rs2171427	A	G	-0.0165	0.0028	4.26E-09	34.502
	rs2245133	C	T	-0.0213	0.0027	7.80E-15	60.384
	rs2297991	T	C	-0.0128	0.0023	1.57E-08	31.970
	rs2398113	G	A	-0.0118	0.0021	1.10E-08	32.657
	rs2494429	A	G	0.0148	0.0027	2.80E-08	30.839
	rs2595644	T	G	-0.0123	0.0021	4.97E-09	34.200
	rs2710651	G	A	0.0116	0.0020	1.23E-08	32.441
	rs2756119	A	G	0.0121	0.0021	8.71E-09	33.109
	rs2807834	T	G	0.0151	0.0022	5.66E-12	47.444
	rs28435470	G	A	0.0119	0.0021	3.29E-08	30.526
	rs2847500	A	G	-0.0225	0.0031	2.77E-13	53.363
	rs290400	G	A	0.0131	0.0022	1.41E-09	36.649
	rs296381	C	T	0.0611	0.0028	2.30E-108	489.048
	rs3114045	T	C	0.0222	0.0030	1.00E-13	55.363

	rs325393	T	G	-0.0136	0.0023	2.03E-09	35.943
	rs34186890	G	A	-0.0157	0.0023	1.33E-11	45.773
	rs34726834	T	C	0.0140	0.0023	2.42E-09	35.600
	rs35270497	T	C	0.0157	0.0027	5.08E-09	34.160
	rs35823191	C	T	-0.0233	0.0021	1.65E-27	118.095
	rs3732220	A	G	-0.0478	0.0036	1.31E-39	173.437
	rs3924150	C	T	-0.0113	0.0021	4.82E-08	29.789
	rs4147536	A	C	0.0148	0.0025	2.76E-09	35.345
	rs4355358	T	C	-0.0259	0.0027	9.75E-22	91.767
	rs4364259	A	G	0.0172	0.0026	1.86E-11	45.118
	rs4420638	G	A	-0.0193	0.0027	3.95E-13	52.667
	rs4580037	C	A	-0.0136	0.0023	1.68E-09	36.310
	rs4694423	A	C	-0.0937	0.0021	1.00E-200	2076.29
	rs4846913	C	A	-0.0132	0.0021	2.19E-10	40.291
	rs4944884	C	T	-0.0714	0.0023	1.00E-200	967.496
	rs512083	C	T	0.0122	0.0020	2.23E-09	35.766
	rs532436	A	G	-0.0151	0.0026	6.00E-09	33.834
	rs5770794	T	C	-0.0133	0.0022	1.74E-09	36.247
	rs6129648	G	A	0.0141	0.0021	2.44E-11	44.579
	rs61698755	T	C	0.0115	0.0021	2.25E-08	31.263
	rs61747728	T	C	0.0303	0.0053	8.83E-09	33.084
	rs62007299	G	A	0.0124	0.0022	3.12E-08	30.632
	rs62012766	C	T	-0.0161	0.0028	6.32E-09	33.735
	rs6600876	G	T	-0.0312	0.0020	3.94E-53	235.412
	rs6834488	T	C	-0.0145	0.0021	2.26E-12	49.247
	rs7122671	A	G	-0.0639	0.0044	1.26E-48	214.752
	rs71601787	A	G	0.0448	0.0022	3.97E-94	423.622
	rs727857	G	A	0.0121	0.0021	9.27E-09	32.989
	rs73413596	C	T	0.0223	0.0039	9.15E-09	33.014
	rs742493	C	T	0.0184	0.0032	1.04E-08	32.769
	rs7528419	G	A	0.0215	0.0024	8.17E-19	78.460
	rs7569755	A	G	0.0136	0.0023	1.49E-09	36.544
	rs7580771	T	G	-0.0166	0.0027	5.15E-10	38.618
	rs7652808	T	G	0.0213	0.0021	1.36E-23	100.229
	rs7657132	G	A	-0.0136	0.0022	6.99E-10	38.023
	rs7712001	G	T	0.0119	0.0021	7.05E-09	33.520

	rs77532868	T	C	0.0260	0.0046	1.28E-08	32.357
	rs77924615	A	G	-0.0152	0.0026	3.94E-09	34.652
	rs77960347	G	A	-0.0526	0.0091	6.53E-09	33.669
	rs804281	A	G	-0.0159	0.0021	1.20E-14	59.539
	rs9375037	C	A	0.0117	0.0021	1.21E-08	32.472
	rs9409266	G	A	0.0168	0.0029	1.24E-08	32.419
	rs9847248	G	A	0.0123	0.0022	4.19E-08	30.059
	rs986649	G	A	0.0129	0.0022	3.51E-09	34.880
	rs9946771	T	C	-0.0234	0.0041	9.47E-09	32.947
Vitamin C	rs10051765	C	T	0.039	0.007	3.64E-09	31.04
	rs10136000	A	G	0.04	0.007	1.33E-08	32.65
	rs117885456	A	G	0.078	0.012	1.70E-11	42.25
	rs13028225	T	C	0.102	0.009	2.38E-30	128.44
	rs174547	C	T	0.036	0.007	3.84E-08	26.45
	rs2559850	A	G	0.058	0.006	6.30E-20	93.44
	rs33972313	C	T	0.36	0.018	4.61E-90	400.00
	rs56738967	C	G	0.041	0.007	7.62E-10	34.31
	rs6693447	T	G	0.039	0.006	6.25E-10	42.25
	rs7740812	G	A	0.038	0.006	1.88E-09	40.11
	rs9895661	T	C	0.063	0.008	1.05E-14	62.02
Vitamin B_6	rs1256335	A	G	-0.14	0.02	1.40E-15	219.04
Vitamin B_{12}	rs1131603	C	T	0.222	0.015	2.11E-48	100.00
	rs1801222	G	A	0.119	0.007	7.24E-74	43.56
	rs34324219	C	A	0.235	0.011	2.54E-109	57.33
	rs41281112	C	T	0.181	0.015	4.60E-34	219.04
folate	rs652197	C	T	0.069	0.01	5.73E-13	203.06
	rs1801133	G	A	0.114	0.008	6.65E-53	47.61
Vitamin E	rs10245705	T	C	0.0663	0.0127	1.95E-07	27.25
	rs11145330	C	A	0.0324	0.0068	1.95E-06	22.70
	rs1404410	G	C	0.0236	0.0052	4.57E-06	20.59
	rs2074731	A	C	0.0184	0.0039	2.31E-06	22.25
	rs7238006	C	T	0.0281	0.0057	6.77E-07	24.30

Supplementary Table 2. Instrumental variables for secondary analysis screening

Micro nutrient	SNPs	EA	OA	BETA	SE	P-vale	F
Vitamin D	rs10014468	A	G	0.01968	0.004177	3.00E-06	22.19838

	rs10118061	A	G	0.012277	0.002461	1.00E-06	24.88635
	rs10146891	T	C	0.010189	0.002071	1.00E-06	24.20488
	rs10161491	C	T	-0.009513	0.001956	1.00E-06	23.6536
	rs10176324	G	A	0.025441	0.00536	2.00E-06	22.52884
	rs10184054	G	C	0.011322	0.002327	1.00E-06	23.67301
	rs10250582	T	C	0.009635	0.001991	1.00E-06	23.4186
	rs10413905	C	T	-0.010308	0.002079	1.00E-06	24.58328
	rs1048940	T	C	-0.016142	0.003292	1.00E-06	24.04335
	rs10814274	T	C	0.009767	0.001969	1.00E-06	24.60543
	rs10860218	C	T	-0.014307	0.002913	1.00E-06	24.12216
	rs10896045	G	A	0.010475	0.002121	1.00E-06	24.39084
	rs11016566	A	G	0.044432	0.009664	4.00E-06	21.13868
	rs11061056	A	G	-0.026534	0.005509	1.00E-06	23.1985
	rs11122449	T	C	0.010888	0.002242	1.00E-06	23.58441
	rs112178027	T	C	-0.012129	0.002636	4.00E-06	21.17187
	rs11249058	A	G	-0.01079	0.002237	1.00E-06	23.26542
	rs112960862	T	C	-0.021421	0.00448	2.00E-06	22.86249
	rs113780007	A	G	0.025704	0.005172	1.00E-06	24.69929
	rs114673443	T	G	0.037046	0.007658	1.00E-06	23.40194
	rs11537654	T	C	-0.010995	0.002222	1.00E-06	24.48513
	rs11592540	A	G	0.011777	0.002397	1.00E-06	24.13978
	rs11612819	C	G	0.010143	0.002096	1.00E-06	23.41803
	rs11624558	C	T	-0.010546	0.002133	1.00E-06	24.44521
	rs11625746	C	T	-0.009389	0.002045	4.00E-06	21.0791
	rs116265747	T	C	0.019272	0.003996	1.00E-06	23.25962
	rs11631408	G	A	0.009219	0.001955	2.00E-06	22.23689
	rs11631567	A	G	0.01293	0.002741	2.00E-06	22.25251
	rs11673587	C	T	0.012326	0.002563	2.00E-06	23.12848
	rs1168100	C	T	-0.010636	0.002129	1.00E-06	24.95774
	rs116962777	A	G	0.036245	0.007261	1.00E-06	24.91744
	rs117048193	C	T	0.040754	0.008831	4.00E-06	21.29711
	rs118147862	A	G	0.023875	0.00475	1.00E-06	25.26385
	rs11822160	A	T	-0.016352	0.003276	1.00E-06	24.9146
	rs12027616	T	C	0.009423	0.001956	1.00E-06	23.20816
	rs12078655	G	A	0.011663	0.002378	1.00E-06	24.05453
	rs12100917	G	A	-0.01398	0.002927	2.00E-06	22.81229

	rs12147588	C	T	0.016914	0.003385	1.00E-06	24.96751
	rs12295437	C	T	-0.022937	0.004733	1.00E-06	23.48552
	rs1230023	G	A	-0.011135	0.002331	2.00E-06	22.81896
	rs12458814	G	A	0.012948	0.002809	4.00E-06	21.24721
	rs1247708	C	T	-0.012351	0.002462	1.00E-06	25.16681
	rs12493498	A	G	0.013908	0.002845	1.00E-06	23.89818
	rs12616915	A	C	-0.015107	0.003072	1.00E-06	24.18321
	rs12620004	C	T	-0.009817	0.002083	2.00E-06	22.21156
	rs12670798	C	T	-0.011317	0.002262	1.00E-06	25.03096
	rs12816349	A	G	0.010442	0.002273	4.00E-06	21.10418
	rs12939053	A	C	0.010519	0.002167	1.00E-06	23.56303
	rs12958051	T	C	-0.011041	0.002206	1.00E-06	25.04989
	rs13011875	T	C	0.010093	0.002021	1.00E-06	24.94066
	rs13020391	T	C	-0.009752	0.002023	1.00E-06	23.2378
	rs13130892	T	C	-0.018906	0.004032	3.00E-06	21.98661
	rs13148767	G	A	0.009951	0.00202	1.00E-06	24.26782
	rs1320782	T	C	0.009942	0.001983	1.00E-06	25.13634
	rs1324190	C	T	-0.009448	0.002016	3.00E-06	21.96336
	rs13263105	G	C	0.010253	0.002055	1.00E-06	24.89306
	rs13409011	A	G	0.010621	0.002112	1.00E-06	25.28966
	rs1384687	A	G	-0.0145	0.002895	1.00E-06	25.08643
	rs140091898	A	G	0.039429	0.008177	1.00E-06	23.2511
	rs141192319	A	G	0.027189	0.005629	1.00E-06	23.3305
	rs141685696	A	C	0.017238	0.003577	1.00E-06	23.22394
	rs142889360	T	C	0.012114	0.002577	3.00E-06	22.09766
	rs145347194	C	T	-0.017004	0.003496	1.00E-06	23.65698
	rs146417206	A	G	0.029057	0.005792	1.00E-06	25.16775
	rs146635149	A	G	0.015677	0.003324	2.00E-06	22.24354
	rs1481024	T	G	0.013356	0.002689	1.00E-06	24.67012
	rs148899426	A	G	0.022343	0.004503	1.00E-06	24.61949
	rs150841811	A	T	0.017652	0.003713	2.00E-06	22.60154
	rs151179502	G	A	0.019776	0.00427	4.00E-06	21.4497
	rs164637	A	G	0.022017	0.004465	1.00E-06	24.31495
	rs16823270	G	A	0.014982	0.003032	1.00E-06	24.41638
	rs16844825	G	A	-0.011662	0.002469	2.00E-06	22.31022
	rs16886847	C	T	-0.011122	0.002243	1.00E-06	24.5871
	rs17035665	T	C	0.011763	0.002422	1.00E-06	23.58783

	rs1704151	G	T	-0.016513	0.003474	2.00E-06	22.59396
	rs17526367	T	C	0.011666	0.002365	1.00E-06	24.33222
	rs17712208	A	T	-0.02633	0.005374	1.00E-06	24.00526
	rs17886395	G	C	-0.014336	0.002878	1.00E-06	24.81272
	rs1843096	A	G	0.012206	0.002432	1.00E-06	25.1895
	rs1854301	T	G	0.01031	0.002219	3.00E-06	21.58752
	rs185575165	A	T	-0.010959	0.002266	1.00E-06	23.38956
	rs1868590	A	C	-0.01463	0.003025	1.00E-06	23.39041
	rs1871462	T	G	-0.009725	0.002022	2.00E-06	23.1322
	rs1879878	G	A	0.00952	0.001977	1.00E-06	23.18785
	rs188265733	T	C	-0.035257	0.00725	1.00E-06	23.6491
	rs1931814	G	A	-0.009103	0.001953	3.00E-06	21.72524
	rs193261074	T	G	0.020329	0.004326	3.00E-06	22.08305
	rs200640263	A	G	-0.015461	0.003176	1.00E-06	23.69814
	rs201920988	A	C	0.010169	0.002089	1.00E-06	23.69625
	rs2023273	G	T	-0.009424	0.001951	1.00E-06	23.33222
	rs2157829	G	A	-0.010282	0.002063	1.00E-06	24.84029
	rs2173201	A	C	-0.011579	0.002317	1.00E-06	24.97411
	rs2229857	C	T	-0.009803	0.002107	3.00E-06	21.64656
	rs2252520	T	A	0.010666	0.002171	1.00E-06	24.13701
	rs2301179	G	A	0.009051	0.001945	3.00E-06	21.65479
	rs2417961	G	A	0.011841	0.002377	1.00E-06	24.81524
	rs2446066	T	G	-0.012598	0.002551	1.00E-06	24.38834
	rs2585433	G	C	0.014209	0.002836	1.00E-06	25.10236
	rs2608949	T	C	-0.011293	0.00232	1.00E-06	23.69423
	rs26579	C	G	0.009993	0.001995	1.00E-06	25.09031
	rs271831	G	A	-0.009819	0.001985	1.00E-06	24.46885
	rs2737245	T	G	0.010811	0.002164	1.00E-06	24.95843
	rs2741044	A	G	0.010452	0.00213	1.00E-06	24.07906
	rs2823021	A	G	-0.011178	0.002407	3.00E-06	21.56632
	rs28895061	T	C	-0.012771	0.002634	1.00E-06	23.50814
	rs289714	A	G	-0.012134	0.002606	3.00E-06	21.67999
	rs2908806	A	C	0.012927	0.002751	3.00E-06	22.08078
	rs2912332	G	A	-0.01631	0.003535	4.00E-06	21.28772
	rs2946405	G	A	-0.01063	0.002188	1.00E-06	23.60325
	rs2977269	G	A	-0.011226	0.002261	1.00E-06	24.65182

	rs2993263	T	C	0.009781	0.001996	1.00E-06	24.01295
	rs34040779	C	T	-0.017714	0.003774	3.00E-06	22.03076
	rs34251103	G	A	0.010504	0.002166	1.00E-06	23.51757
	rs34311816	T	C	-0.010329	0.002249	4.00E-06	21.09297
	rs34329	C	G	-0.010474	0.002111	1.00E-06	24.61777
	rs34368067	A	G	0.023141	0.00476	1.00E-06	23.63471
	rs34699226	G	A	-0.011595	0.002337	1.00E-06	24.61637
	rs35456421	T	C	0.015258	0.003131	1.00E-06	23.74811
	rs35634730	T	C	0.013975	0.002862	1.00E-06	23.84319
	rs36099109	T	C	-0.012305	0.002576	2.00E-06	22.81768
	rs4078354	G	A	-0.010205	0.002061	1.00E-06	24.51715
	rs41302867	A	G	0.014003	0.002965	2.00E-06	22.30451
	rs4505147	A	C	-0.01032	0.002065	1.00E-06	24.97579
	rs4612354	C	T	-0.010758	0.002208	1.00E-06	23.73914
	rs4619804	C	A	-0.01095	0.002221	1.00E-06	24.30699
	rs4626538	T	G	0.009085	0.001962	4.00E-06	21.44134
	rs4686529	G	A	-0.009163	0.001987	4.00E-06	21.2657
	rs4692473	A	C	-0.00973	0.002089	3.00E-06	21.69446
	rs4711968	C	T	0.010254	0.002123	1.00E-06	23.32849
	rs4722620	T	C	0.009775	0.002123	4.00E-06	21.19989
	rs4793915	T	C	-0.009145	0.001963	3.00E-06	21.70335
	rs4865160	A	G	-0.010184	0.002072	1.00E-06	24.15779
	rs4970924	G	A	0.009349	0.001939	1.00E-06	23.24742
	rs4971866	C	G	-0.013101	0.002698	1.00E-06	23.57898
	rs4980634	C	T	-0.00967	0.001983	1.00E-06	23.77976
	rs500328	C	T	0.009339	0.002005	3.00E-06	21.69562
	rs523518	G	A	-0.009757	0.001984	1.00E-06	24.18518
	rs548972072	G	C	-0.011147	0.002389	3.00E-06	21.77127
	rs55728843	T	C	-0.015346	0.003181	1.00E-06	23.27357
	rs57601828	T	A	0.009742	0.002023	1.00E-06	23.1902
	rs57766735	T	A	0.052241	0.010692	1.00E-06	23.87289
	rs58114349	C	T	0.014169	0.00292	1.00E-06	23.54576
	rs58725556	G	A	0.016528	0.003425	1.00E-06	23.28732
	rs6016381	C	T	0.009706	0.002011	1.00E-06	23.29466
	rs60566117	A	G	-0.011521	0.00232	1.00E-06	24.66064
	rs6068773	T	C	-0.009773	0.001948	1.00E-06	25.16969
	rs62011267	T	C	-0.011039	0.002246	1.00E-06	24.15683

	rs62011683	C	T	-0.021718	0.004722	4.00E-06	21.15377
	rs62078461	A	G	0.028409	0.006179	4.00E-06	21.13857
	rs62109667	G	A	-0.011125	0.002241	1.00E-06	24.64429
	rs62261725	G	A	-0.009571	0.002073	4.00E-06	21.31651
	rs62308866	G	A	0.010071	0.002071	1.00E-06	23.64749
	rs62471929	G	A	0.021357	0.00438	1.00E-06	23.77564
	rs62493792	A	T	0.011249	0.002313	1.00E-06	23.65247
	rs62493995	G	T	-0.010929	0.002225	1.00E-06	24.12686
	rs62530961	T	C	-0.015615	0.003303	2.00E-06	22.34945
	rs6438867	C	T	-0.009913	0.002053	1.00E-06	23.31483
	rs651007	T	C	-0.011425	0.00241	2.00E-06	22.47389
	rs667351	A	G	0.01401	0.002861	1.00E-06	23.97952
	rs6700772	G	C	0.009784	0.001974	1.00E-06	24.56623
	rs6921141	G	A	0.010178	0.002064	1.00E-06	24.31675
	rs71615088	C	T	-0.012937	0.002626	1.00E-06	24.27044
	rs7251312	T	C	0.012375	0.002612	2.00E-06	22.44626
	rs72681845	T	G	-0.0221	0.00457	1.00E-06	23.3858
	rs72787359	C	A	0.018298	0.003789	1.00E-06	23.32158
	rs72793247	T	C	-0.021439	0.004394	1.00E-06	23.80614
	rs72800804	T	G	-0.025017	0.005103	1.00E-06	24.03363
	rs72866406	C	T	-0.046749	0.009576	1.00E-06	23.83287
	rs73030358	G	A	-0.013085	0.002635	1.00E-06	24.65961
	rs73178550	C	G	-0.017206	0.003665	3.00E-06	22.04001
	rs7395670	T	C	0.009636	0.001976	1.00E-06	23.78043
	rs742493	C	T	0.015166	0.003077	1.00E-06	24.29333
	rs74886767	G	A	-0.024113	0.005005	1.00E-06	23.21103
	rs75604577	A	C	-0.031542	0.006785	3.00E-06	21.61119
	rs75622376	T	C	0.026071	0.00536	1.00E-06	23.65842
	rs76847001	T	C	0.01004	0.002085	1.00E-06	23.18757
	rs7687826	T	G	0.019378	0.003888	1.00E-06	24.84079
	rs7696851	T	C	0.021805	0.004393	1.00E-06	24.63711
	rs7714980	T	A	0.011603	0.002403	1.00E-06	23.31487
	rs78752474	T	C	0.020403	0.004231	1.00E-06	23.25423
	rs78856761	T	C	-0.02874	0.006011	2.00E-06	22.8602
	rs79496165	G	A	-0.017195	0.003553	1.00E-06	23.42146
	rs8015301	T	C	-0.015226	0.003261	3.00E-06	21.80068

	rs8043085	T	G	-0.011461	0.002302	1.00E-06	24.78759
	rs8045120	T	G	0.009872	0.002026	1.00E-06	23.74277
	rs8048397	T	C	0.011688	0.002395	1.00E-06	23.81603
	rs8086642	A	G	-0.009711	0.001952	1.00E-06	24.74961
	rs8090363	G	A	-0.009867	0.001997	1.00E-06	24.41261
	rs821405	G	A	-0.014652	0.002971	1.00E-06	24.3214
	rs827419	C	A	-0.009418	0.002014	3.00E-06	21.86746
	rs888770	G	T	-0.010671	0.002189	1.00E-06	23.76395
	rs9381174	C	T	0.010832	0.002264	2.00E-06	22.89098
	rs9514994	C	A	-0.011648	0.002499	3.00E-06	21.72552
	rs9537006	C	A	-0.010597	0.002197	1.00E-06	23.26514
	rs9598096	C	T	0.010258	0.002093	1.00E-06	24.02077
	rs9817526	A	G	0.010267	0.002131	1.00E-06	23.21241
	rs9932726	G	A	0.009882	0.002017	1.00E-06	24.00368
	rs9940346	C	T	-0.009812	0.00199	1.00E-06	24.31134
Vitamin E	rs111306778	A	G	-0.0479658	0.00957227	5.40E-07	25.10924
	rs12165526	A	T	0.0479017	0.00917775	1.80E-07	27.2414
	rs12421920	G	A	-0.0434479	0.00938496	3.70E-06	21.43249
	rs12899673	A	G	0.0268951	0.00581803	3.80E-06	21.3695
	rs2723979	G	T	-0.0266142	0.00552745	1.50E-06	23.1834
	rs35218694	G	A	-0.0741637	0.0153104	1.30E-06	23.46441
	rs4903544	T	C	-0.0295098	0.0060211	9.50E-07	24.02043
	rs536912	A	C	0.0304596	0.00620091	9.00E-07	24.1289
	rs6033	G	A	-0.0516563	0.0105563	9.90E-07	23.94546
	rs71385328	G	A	0.130015	0.0262064	7.00E-07	24.61343
	rs79966958	T	C	-0.116527	0.024505	2.00E-06	22.61225
	rs979218	C	A	-0.0430325	0.00922259	3.10E-06	21.77145
Vitamin C	rs114598078	T	C	0.0655782	0.0137636	1.90E-06	22.70152
	rs11650824	A	T	0.0794787	0.0158822	5.60E-07	25.04264
	rs17482258	T	C	0.042829	0.00926045	3.70E-06	21.39004
	rs1883993	A	G	0.044959	0.00935324	1.50E-06	23.10517
	rs2018201	G	T	-0.0808079	0.0171646	2.50E-06	22.1636
	rs4238567	C	T	0.0253068	0.00550812	4.30E-06	21.109
	rs4481190	C	A	-0.0306375	0.00574377	9.60E-08	28.45198
	rs61868302	T	C	-0.0571013	0.0118391	1.40E-06	23.26241
	rs7626478	A	G	0.0279796	0.00610134	4.50E-06	21.02967
	rs9540734	A	G	-0.0259285	0.00548469	2.30E-06	22.34862

Vitamin B_{12}	rs10924919	T	C	-0.0286276	0.0056423	3.90E-07	25.7429
	rs112961770	C	G	-0.0888494	0.0184324	1.40E-06	23.23514
	rs12776611	A	G	-0.0878135	0.0188958	3.40E-06	21.59693
	rs1419875	G	T	-0.0315231	0.00683151	3.90E-06	21.29239
	rs148901823	G	A	-0.0486291	0.0100516	1.30E-06	23.40572
	rs388561	C	T	0.040271	0.00854945	2.50E-06	22.18751
	rs6088761	G	A	0.0292724	0.00627081	3.00E-06	21.79061
	rs61994378	C	T	0.0934555	0.0197741	2.30E-06	22.33656
	rs67568068	C	T	-0.0317378	0.00664671	1.80E-06	22.80028
Vitamin B_6	rs10138490	C	T	-0.0534963	0.0114138	2.80E-06	21.96782
	rs12226112	T	G	0.0283412	0.00572659	7.50E-07	24.49313
	rs12412051	C	G	0.0708635	0.0149768	2.20E-06	22.38758
	rs141933624	A	G	-0.0897347	0.0192811	3.30E-06	21.65994
	rs155599	C	T	0.0342533	0.00598463	1.00E-08	32.75897
	rs183178622	T	C	-0.0989483	0.0206841	1.70E-06	22.8846
	rs188211816	A	G	-0.0786243	0.0162809	1.40E-06	23.32152
	rs361294	C	A	-0.0273612	0.00595181	4.30E-06	21.13354
	rs3745438	C	T	-0.071266	0.0155965	4.90E-06	20.87904
	rs3772928	C	T	-0.0292449	0.00553405	1.30E-07	27.92635
	rs67450584	T	C	0.036705	0.00745732	8.60E-07	24.22618
	rs7205927	C	A	-0.0257861	0.00553457	3.20E-06	21.70719
	rs77806858	C	T	-0.0505124	0.0106104	1.90E-06	22.66379
	rs9560457	T	C	0.0255037	0.00553964	4.10E-06	21.19548
Folate	rs139588363	C	T	0.05368	0.0116503	4.10E-06	21.23004
	rs148031795	T	C	0.104439	0.0223794	3.10E-06	21.77853
	rs1502443	G	C	0.0258943	0.00563529	4.30E-06	21.11426
	rs16956822	A	G	-0.0792943	0.017182	3.90E-06	21.29789
	rs2449166	T	C	0.0252219	0.00546681	4.00E-06	21.28569
	rs3772928	C	T	-0.0272574	0.00552739	8.20E-07	24.31804
	rs57185514	G	A	-0.138679	0.0301895	4.40E-06	21.10132
	rs7074988	G	A	-0.051278	0.0111147	4.00E-06	21.28466
	rs76630415	G	T	-0.0373906	0.00670304	2.40E-08	31.11582
	rs76802001	A	G	-0.0677158	0.0147765	4.60E-06	21.00085
	rs78074774	T	C	0.0601471	0.0131667	4.90E-06	20.86774
	rs79748722	T	C	-0.0756746	0.0164864	4.40E-06	21.06923
	rs79975477	T	C	0.0731991	0.0156345	2.80E-06	21.92015

	rs8085166	G	A	0.0278138	0.00581169	1.70E-06	22.90423

Supplementary Table 3. Association circulating micronutrient levels-related with frailty using heterogeneity test in main analysis.

Exposures	Outcome	No.of Ivs	Heterogeneity	
			Q	P-Cochran's
Vitamin E	Frailty	5	0.654	0.956
Vitamin D		101	214.96	2.14E-10
Vitamin C		11	28.792	0.001
Vitamin B_{12}		4	10.733	0.15
Vitamin B_6		1	0.832	0.361
folate		2	0.888	0.345

Supplementary Table 4. Results of pleiotropy tests for main analysis

Exposures	Outcome	MR-Egger		MR-PRESSO	
		P-value	Intercept	No. of outlier	*P*
Vitamin E	Frailty	0.677	-0.003	NA	0.29
Vitamin D		0.583	-0.0005	NA	0.85
Vitamin C		0.744	-0.0011	NA	0.49
Vitamin B_{12}		0.149	0.005	NA	0.68
Vitamin B_6		\	\	\	\
folate		\	\	\	\

Supplementary Table 5. Association circulating micronutrient levels-related with frailty using heterogeneity test in secondary analysis.

Exposures	Outcome	No.of Ivs	Heterogeneity	
			Q	P-Cochran's
Vitamin E	Frailty	12	23.015	0.017
Vitamin D		198	363.67	4.99E-12
Vitamin C		10	13.962	0.123
Vitamin B12		9	4.696	0.789
Vitamin B6		14	17.816	0.164
folate		14	21.366	0.065

Supplementary Table 6. Results of pleiotropy tests for secondary analysis

Exposures	Outcome	MR-Egger		MR-PRESSO	
		P-value	Intercept	No. of outlier	*P*
Vitamin E	Frailty	0.724	-0.001	NA	0.55
Vitamin D		0.118	-0.0022	NA	0.0004
Vitamin C		0.379	-0.0045	NA	0.10
Vitamin B_{12}		0.915	-0.0004	NA	0.27
Vitamin B_6		0.033	0.007	NA	0.33
folate		0.599	-0.002	NA	0.17
